# Using deep-learning predictions of inter-residue distances for model validation

**DOI:** 10.1107/S2059798322010415

**Published:** 2022-11-25

**Authors:** Filomeno Sánchez Rodríguez, Grzegorz Chojnowski, Ronan M. Keegan, Daniel J. Rigden

**Affiliations:** a Institute of Systems, Molecular and Integrative Biology, University of Liverpool, Liverpool L69 7ZB, United Kingdom; b Life Science, Diamond Light Source, Harwell Science and Innovation Campus, Didcot OX11 0DE, United Kingdom; c European Molecular Biology Laboratory, Hamburg Unit, Notkestrasse 85, 22607 Hamburg, Germany; d UKRI–STFC, Rutherford Appleton Laboratory, Research Complex at Harwell, Didcot OX11 0FA, United Kingdom; MRC Laboratory of Molecular Biology, United Kingdom

**Keywords:** model validation, inter-residue distances, *AlphaFold*2, *ConKit*, *conkit-validate*

## Abstract

Determination of protein structures typically entails building a model that satisfies the collected experimental observations and its deposition in the Protein Data Bank. Experimental limitations can lead to unavoidable uncertainties during the process of model building, which result in the introduction of errors into the deposited model. Many metrics are available for model validation, but most are limited to consideration of the physico-chemical aspects of the model or its match to the experimental data. The latest advances in the field of deep learning have enabled the increasingly accurate prediction of inter-residue distances, an advance which has played a pivotal role in the recent improvements observed in the field of protein *ab initio* modelling. Here, new validation methods are presented based on the use of these precise inter-residue distance predictions, which are compared with the distances observed in the protein model. Sequence-register errors are particularly clearly detected and the register shifts required for their correction can be reliably determined. The method is available in the *ConKit* package (https://www.conkit.org).

## Abstract 2

The use of *AlphaFold*2 predictions for the detection and correction of sequence-register errors among protein structures determined using cryo-EM deposited in the Protein Data Bank is described.

## 1. Introduction

Structural determination of proteins may be carried out using a range of different techniques, of which macromolecular X-ray crystallography (MX) and cryogenic electron microscopy (cryo-EM) are currently the most popular. These experiments typically culminate in the creation of a model that satisfies the experimental observations collected, and which is subsequently deposited in the Protein Data Bank (PDB; Berman *et al.*, 2000 ▸). However, as in all experiments, these observations will have unavoidable uncertainties caused by experimental limitations, which can result in the introduction of errors into the final model.

Such errors were particularly common during the early stages of X-ray crystallography, when technical advances allowed an increasing number of protein folds to be experimentally determined and deposited in the PDB. It was also during this time that some deposited structures were first found to contain major errors, highlighting the need for model validation tools (Hooft *et al.*, 1996 ▸; MacArthur *et al.*, 1994 ▸; Kleywegt & Jones, 1995 ▸). Several computational methods and systems were developed to address the issue. Among them were *PROCHECK* (Laskowski *et al.*, 1993 ▸) and *WHATIF* (Vriend, 1990 ▸), each majoring on geometric and stereochemical properties and generating residue-by-residue reports to inform the user of potential errors in the model. However, it was noted that stereochemical analyses could be insufficient for the unambiguous identification of errors, leading to the introduction of methods such as *VERIFY*_3*D* (Lüthy *et al.*, 1992 ▸) based on statistics of favoured amino-acid environments, *ProSA* (Sippl, 1993 ▸) based on the combination of a C^β^–C^β^ (or C^α^–C^α^) potential and solvent-exposure statistics, *DACA* (Vriend & Sander, 1993 ▸) based on the evaluation of interatomic contacts to test protein model packing and *ERRAT* (Colovos & Yeates, 1993 ▸), which is based on the statistics of nonbonded interactions between C, N and O atoms. Further notable contributions include *Coot* (Emsley *et al.*, 2010 ▸), which provided interactive model-building tools coupled with a series of residue-by-residue model validation metrics based both on the geometric properties of the model and its match to the map. *MolProbity* (Davis *et al.*, 2007 ▸) was later released and provided validation reports based on the analysis of all-atom contacts together with other geometric and dihedral angle analyses.

With the recent technical improvements in image processing and electron detectors, a rapid increase in the number of molecules deposited in the PDB which were solved using cryo-EM has been observed in recent years (Chiu *et al.*, 2021 ▸). Compared with X-ray crystallography, these models are often built using lower resolution data, with maps that have varying levels of local resolution at different parts of the model, all of which can hinder model building and make cryo-EM models more susceptible to errors. The discovery of modelling errors among cryo-EM structures recently deposited in the PDB (Croll *et al.*, 2021 ▸; Chojnowski *et al.*, 2022 ▸; Weiss *et al.*, 2016 ▸) has highlighted the need for new tools for model validation (Lawson & Chiu, 2018 ▸; Afonine *et al.*, 2018 ▸). This has led to the creation of sophisticated new tools for model validation, such as *checkMySequence* (Chojnowski, 2022 ▸), which uses the latest advances in machine learning for the detection of out-of-register sequence errors. New metrics for the assessment of the quality of a model and its fit to the map have also been introduced in recent years, such as SMOC (Joseph *et al.*, 2016 ▸), a segment-based Manders’ overlap coefficient between the model and the map, FSC-Q (Ramírez-Aportela *et al.*, 2021 ▸), a model-quality validation score based on the local Fourier shell correlation between the model and the map, and the map Q-score (Pintilie *et al.*, 2020 ▸), which measures atomic resolvability. The creation of these metrics has resulted in the development of new user interfaces to integrate these different metrics in order to facilitate their interpretability and to provide an all-in-one package. This is the case for the *CCP-EM* validation task (Joseph *et al.*, 2022 ▸), which combines several of these new metrics and tools into a graphical user interface. Further developments in the interpretability of validation metrics came with the release of *Iris* (Rochira & Agirre, 2021 ▸), a tool that combines different validation metrics calculated on a residue-by-residue basis.

Recent developments in the field of evolutionary covariance and machine learning have enabled the precise prediction of residue–residue contacts and increasingly accurate inter-residue distance predictions (Ruiz-Serra *et al.*, 2021 ▸). Access to this accurate covariance information has played an essential role in the latest advances observed in the field of protein structural bioinformatics, particularly the improvement of protein *ab initio* modelling, with the most notable examples being *AlphaFold*2 (Jumper *et al.*, 2021 ▸) and *RoseTTAFold* (Baek *et al.*, 2021 ▸).

Here, we present new validation methods based on the availability of accurate inter-residue distance predictions. Potential errors are recognized as residues and regions for which the contacts and inter-residue distances observed in the model differ significantly from those predicted by deep learning-based methods. A series of metrics relating to the consistency of observed and predicted contacts and distances are fed into a support-vector machine classifier that was trained to detect model errors using historical data from the EM Validation Challenges (Lawson *et al.*, 2021 ▸; Lawson & Chiu, 2018 ▸). Further detection of possible register errors is specifically performed by performing an alignment of the predicted contact map and the map inferred from the contacts observed in the model. Regions of the model in which the maximum contact overlap is achieved through a sequence register different to that observed in the model are flagged and the optimal sequence register can then be used to fix the register error. The results suggest that the detection of model errors and the correction of sequence-register errors is possible through the use of the trained classifier in conjunction with the contact-map alignment, as revealed by analysing a set of structures deposited in the PDB. This approach, which is implemented in *ConKit* (Simkovic *et al.*, 2017 ▸) through the command-line option *conkit-validate*, thus provides a new tool for protein structure validation that is orthogonal to existing methods.

## 2. Materials and methods

### 2.1. Creation of a training data set of misregistered residues extracted from the EM modelling challenges

Structures submitted to the EM modelling challenges that took place in 2016, 2019 and 2021 (Lawson *et al.*, 2021 ▸; Lawson & Chiu, 2018 ▸) were analysed in order to create a database containing modelling errors annotated according to whether the cause was or was not an incorrect sequence register. Firstly, structures in which more than half of residues scored a sequence-dependent local–global alignment (LGA; Zemla, 2003 ▸) above 8 Å between the target and the experimentally determined structure were discarded. For the remaining structures, LGA values were smoothed using a three-residue window rolling average. Model regions in which at least three consecutive residues scored a smoothed LGA value of 3 Å or higher were then visually inspected, searching for register errors. These errors were defined as ranges of residues where despite having a smoothed LGA above 3 Å, the main chain had been modelled correctly when compared with the ground-truth solution. To reduce redundancy, register errors found within the same sets of residues across different models submitted for the same target were removed, except for the error affecting the largest number of residues, which was selected as the representative error. All residues found among the resulting set of register errors were then labelled with the positive class (modelled incorrectly) and taken into a database of register errors. The remaining residues found in the models from which these register errors were taken were also added to the database, but they were instead labelled with the negative class (modelled correctly) if the smoothed LGA was below 3 Å; otherwise, they were considered to be part of a modelling error and the positive class was assigned (modelled incorrectly). This resulted in the creation of a data set consisting of 8620 residues, of which 6192 were labelled with the negative class and 2428 with the positive class. Residues labelled with the positive class were extracted from 76 sequence-register errors (2278 residues) and 12 other modelling errors (150 residues).

### 2.2. Prediction of inter-residue distances using *AlphaFold*2

Predictions of inter-residue contacts and distances were obtained for the models being validated using *AlphaFold*2. The original CASP14 model preset was used and the database search was set to full mode. All other parameters were left with their default values. Predictions were carried out on a computing grid in which each node is equipped with a twin 16-core Intel Xeon Gold 5218 running at 2.3 GHz, 160 GB of memory and four NVIDIA Tesla V100 chips with 16 GB of video memory each.

For each case, five models were produced and the inter-residue distance predictions for the model with the highest predicted local distance difference test (pLDDT; Mariani *et al.*, 2013 ▸) were taken. For each residue pair in the structure, this prediction contains the predicted probability that these residues are within a series of distance bins. These distances were processed for all residue pairs so that the midpoint value of the distance bin with the highest probability was considered to be the predicted distance and the probability associated with this bin was considered to be the confidence score. Contact predictions were derived from the distance prediction by adding together all of the the probabilities observed across the distance bins up to 8 Å. The top *L*/2 contacts scoring the highest probability values were then taken to form the final predicted contact maps, where *L* denotes the sequence length.

### 2.3. New covariance-based metrics for model validation and feature engineering

A set of new metrics were developed with the aim of comparing the inter-residue contacts and distances observed in a model and those predicted for the protein of interest. Rather than a global comparison of the similarity of two contact maps or distograms, these metrics were designed for local model validation, hence they are calculated on a residue-by-residue basis.

First, a weighted root-mean-square deviation (wRMSD) of the predicted and the observed inter-residue distances was calculated for each residue of the model as follows,

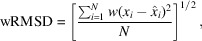

where *N* represents the number of residues in the model, *x* represents the observed distance for a pair of residues, 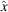 represents the predicted distance for a pair of residues and *w* represents the confidence of the predicted inter-residue distance (a value between 0 and 1).

A series of metrics based on analysis of the inter-residue contacts on a residue-by-residue basis were also defined as follows,

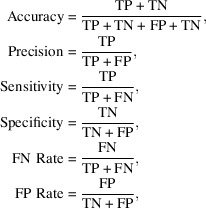

where TP represents true positives, FP represents false positives, TN represents true negatives and FN represents false negatives. Additionally, the raw count of FN and FP was also used as two additional features. Two residues are considered to be in contact with each other when their C^β^ atoms are within 8 Å of each other (C^α^ for glycine). For the calculation of these metrics, the top *L*/2 contacts with the highest confidence values were used, where *L* denotes the number of residues in the protein sequence.

For all of the proposed metrics, smoothed and unsmoothed versions were calculated. Whereas in the unsmoothed version the values observed across the residues of the model were kept intact, in the smoothed version these values were smoothed using a convolution approach. In this approach, a five-point unweighted filter was used to convolve the raw data, making this transformation equivalent to using a moving five-residue window averaging technique, with the added benefit of not losing data at the edges of the model where there is not sufficient information to calculate a window average.

Additionally, a *Z*-score derived metric was computed for all of the proposed metrics. This was calculated by taking the value observed for each residue of the model and using the values observed for the residues within a range of 10 Å as the full sample.

**Figure 1:**
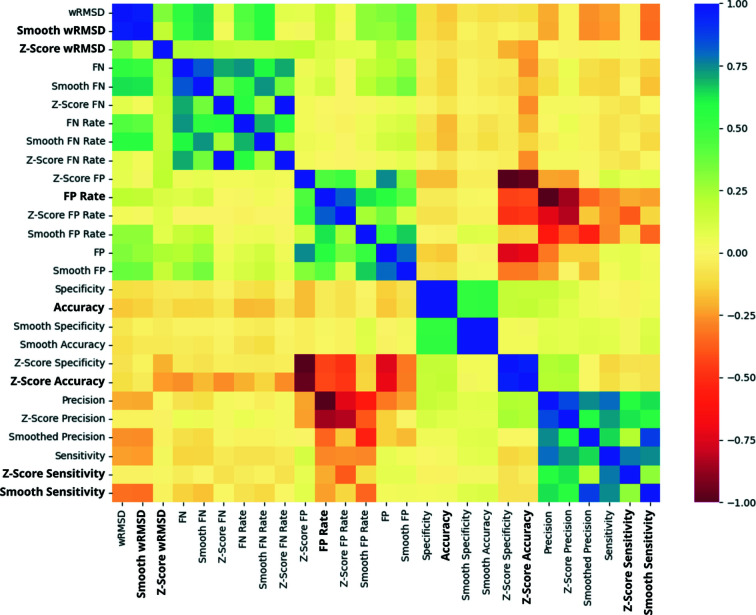
Correlation matrix for the proposed covariance-based metrics for model validation. The colour in each cell corresponds to the Pearson’s correlation coefficient observed between each pair of metrics. The scale goes from red for negative correlation to deep blue for positive correlation. The final set of metrics are highlighted in bold.

This resulted in the creation of 24 metrics: one distance prediction-based metric, seven contact prediction-based metrics, their smoothed and unsmoothed versions, and the additional *Z*-score version. To ensure minimal autocorrelation, Pearson’s correlation coefficients were examined for all possible pairs of these metrics using the values observed across the EM modelling challenge data set described in Section 2.1 (Fig. 1 ▸). Where a pair or a group of metrics shared an absolute correlation value of 0.4 or higher, only one representative metric was taken and the others were discarded. The representative metric was selected based on initial results obtained using a linear discriminant analysis using the residues from the EM challenge data set and setting the class label as the target. For a given set of correlated metrics, the feature with the highest coefficient observed in this analysis was selected as the representative. This resulted in the creation of a final set of seven metrics: Accuracy, FP Rate, smooth Sensitivity, smooth wRMSD, *Z*-score Accuracy, *Z*-score Sensitivity and *Z*-score wRMSD.

### 2.4. Machine-learning training and hyperparameter tuning

Residues found in the data set created by extracting residues from the EM modelling challenge submissions were used to train several machine-learning algorithms for the detection of register errors. For each observation in this data set, the covariance metrics described in Section 2.3 were calculated. Additional features describing the local environment of the residue were also included in the observations, specifically the residue solvent accessibility (ACC) and the secondary-structure element in which the residue was located (helix, β-sheet or coil), which was assigned using *DSSP* (Touw *et al.*, 2015 ▸). Residues found to be part of register and other modelling errors were labelled with the positive target class, or otherwise with the negative class. To create the training and test sets, residues labelled with the positive class were randomly split using a 80:20 ratio. In order to ensure balanced data sets, the same number of residues labelled with the negative class were randomly selected and added to each set. This resulted in the creation of a training set consisting of 3884 observations and a test set formed by 972 observations, with both of them having a balanced number of observations in each class. The data was then standardized to *Z*-scores using a standard scaler, fitted only using the data seen in the training set to prevent data leakage. Optimal training hyperparameters for each classifier were found by performing a random search of 200 iterations using the mean accuracy as the scoring function. All of the algorithm implementations were performed with *scikit-learn* version 0.24.2 (Pedregosa *et al.*, 2011 ▸).

### 2.5. Contact map alignment-based sequence reassignment

The alignment between predicted contact maps and the contact maps observed in the models of interest was calculated using *map_align* (Ovchinnikov *et al.*, 2017 ▸). This tool creates an alignment between two input contact maps so that the contact maximum overlap (CMO) is achieved (Andonov *et al.*, 2011 ▸) by introducing and extending gaps as necessary. The CMO is defined as the number of matching contacts between the two input contact maps when optimal alignment is achieved. If a misalignment between the input contact maps is found for a set of residues, the sequence register used to achieve the CMO between predicted and observed contacts is proposed as a fix for the possible register error.

### 2.6. Creation of filters to reduce the number of contact-map alignment-based false positives

Three criteria were designed to detect and discard register errors predicted using the contact-map alignment-based approach in regions of the model where the contact-map misalignment could have been caused by reasons other than a register error.

The first criterion was to discard register errors where either the mean or the median number of predicted contacts per residue observed across the affected residue range was lower than two contacts. This was performed to discard register errors predicted without sufficient contact information to produce a reliable contact-map alignment. Secondly, predicted register errors affecting residues where the average pLDDT assigned by *AlphaFold*2 was below 65 were discarded. This was performed to remove instances where a register error was predicted based on predictions of insufficient quality. Lastly, for the third criterion, a structural alignment between the deposited model in which a register error was detected and the model predicted by *AlphaFold*2 was performed using *GESAMT* (Krissinel, 2012 ▸). The *GESAMT* Q-score was then calculated for the range of residues affected by the possible register error. Those cases where the Q-score was below 0.5 were discarded to avoid instances with high discrepancy between the deposited model and the predicted model, an indication that the predictions produced by *AlphaFold*2 could be inaccurate or that the predicted model was modelled in a different conformation to that deposited.

### 2.7. Creation of a benchmark data set consisting of PDB-deposited structures solved by cryo-EM

Protein structures determined using cryo-EM at 5 Å resolution or better, with or without a nucleic acid component, were selected from the Protein Data Bank (PDB; Berman *et al.*, 2000 ▸) as of 10 November 2021. For practical reasons, a subset of 5744 with the size of the corresponding compressed EM maps not exceeding 200 MB was selected from 7241 available structures. Next, all structures were automatically analysed using the *checkMySequence* validation tool (Chojnowski, 2022 ▸), which identified 419 chains with tentative sequence-assignment issues in 246 structures that were used for further analysis.

Using the reference sequence deposited in the PDB, distance predictions were obtained for each individual chain. For 55 chains this was not possible due to hardware limitations, particularly the system running out of memory before the predictions could be completed. Ultimately, our analysis could be applied to 364 protein chains found in structures deposited in the PDB where possible register errors were reported by *checkMySequence*. To ensure a match between the residue numbering observed in the deposited models and the numbering in the reference sequence used to obtain the inter-residue distance predictions, *CROPS* (https://github.com/rigdenlab/crops) was used to renumber the models based on the reference. For the 149 cases where this was not possible due to major inconsistencies between the protein sequence and the residue numbering in the deposited model, a manual inspection was carried out.

## 3. Results and discussion

### 3.1. Machine learning detects register errors using covariance-based metrics

**Figure 2:**
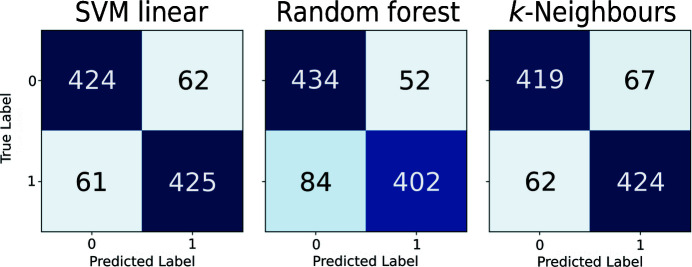
Confusion matrices obtained after predicting the data points in the test set with each of the three classifiers being assessed: support-vector machines, random forest and *k*-nearest neighbours. Residues present in a register error are labelled as class 1 and residues modelled correctly are labelled as class 0. The numbers inside each cell correspond to the number of data points in each category, which is proportional to the colour intensity in each cell.

**Figure 3:**
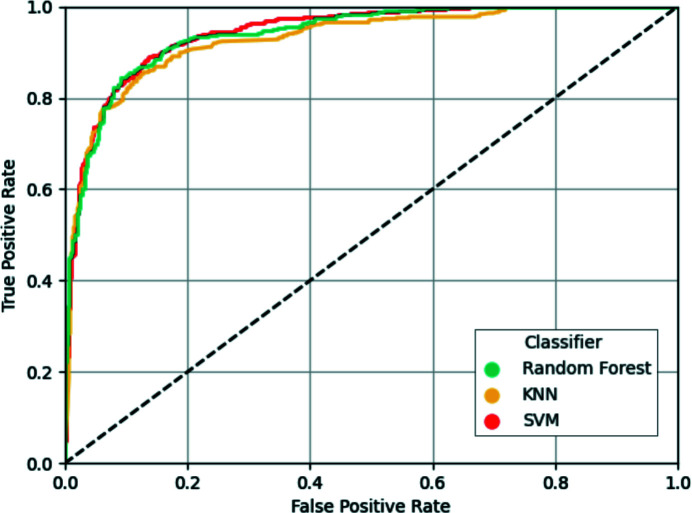
Receiver operating characteristic (ROC) curve depiction of the results obtained on the test set using each of the three classifiers being assessed: support-vector machines (SVM, red), random forest (yellow) and *k*-nearest neighbours (KNN, turquoise). The horizontal and vertical axes correspond to the two operating characteristics, the false-positive rate and true-positive rate, respectively, measured at different confidence threshold values. A dashed diagonal line represents the performance of a no-skill classifier where predictions are made at random.

In order to create a classifier that is able to detect register errors and other modelling errors using the newly developed covariance-based metrics, three different types of classifiers available in *scikit-learn* (Pedregosa *et al.*, 2011 ▸) were selected: support-vector machines using a linear kernel (SVM), random forest (RF) and *k*-nearest neighbours (KNN). To create the training and test sets, residues from the models submitted to the EM modelling challenges were extracted and a train–test split was created as described in Sections 2.1 and 2.4. Optimal training hyperparameters found in a random search were then used to train each of the classifiers using the observations in the training set, and prediction of the test set was then attempted. Analysis of the results obtained after prediction of the test samples with each of the classifiers revealed an overall good performance by all three classifiers, which were able to provide accurate predictions for most residues present in the test set (Fig. 2 ▸). Further analysis was performed by plotting the receiver operating characteristic (ROC) curves of each classifier (Fig. 3 ▸), which showed that the three classifiers performed well at different confidence-score threshold cutoff values.

The performance of the trained classifiers on the hold-out test-set samples was further analysed based on several metrics (Table 1 ▸). Interestingly, only some minor differences were observed across the different classifiers, which had a similar overall good performance at predicting whether or not the residues in the test set were part of model errors. Despite achieving the highest Precision (0.885), the RF classifier was not the classifier of choice to be integrated into *ConKit*; the SVM was instead selected after having scored the highest Accuracy, Area Under the ROC Curve (AUC), Recall and F1-Score.

**Figure 4:**
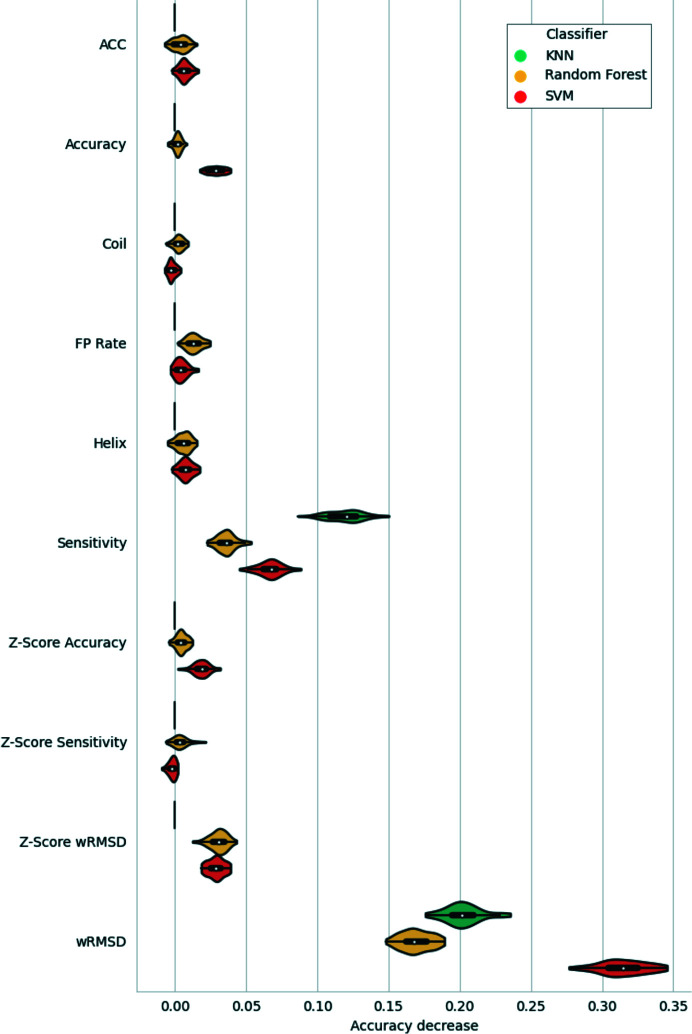
Depiction of the results of the feature-permutation analysis for the three classifiers being assessed: support-vector machines (SVM, red), random forest (yellow) and *k*-nearest neighbours (KNN, turquoise). Each violin depicts the distribution of values observed for the Accuracy decrease recorded after each independent feature permutation. Black bars inside the violins depict the interquartile range, with a white dot showing the median and black whiskers the maximum and minimum quartiles. Note that ‘Accuracy decrease’ here refers to the Accuracy achieved by the trained classifiers with the hold-out test set after feature permutation, while the ‘Accuracy’ shown on the vertical axis refers to the metric used as a predictive feature.

In order to assess the importance of each of the features in the models being compared, a feature-permutation analysis was carried out. In this analysis, the values of each feature were randomly shuffled across the samples in the hold-out test set. Prediction was then attempted with the trained classifiers and the decrease in accuracy with respect to the baseline (the accuracy of the trained classifier in the test set without shuffling) was recorded. Each feature permutation was repeated 50 times at random for consistency of results. Analysis of the results obtained after these permutations (Fig. 4 ▸) revealed a strong decrease in accuracy after the shuffling of the wRMSD values, an indication that all three models depend on wRMSD the most for accurate prediction of whether or not a given residue is part of a model error. This was followed by the Sensitivity, which was the second most important feature across the three classifiers. No major differences were observed for the rest of the features across the different classifiers, with the exception of the permutation of the Accuracy and *Z*-score Accuracy features, which showed a decrease in performance only for the SVM.

**Figure 5:**
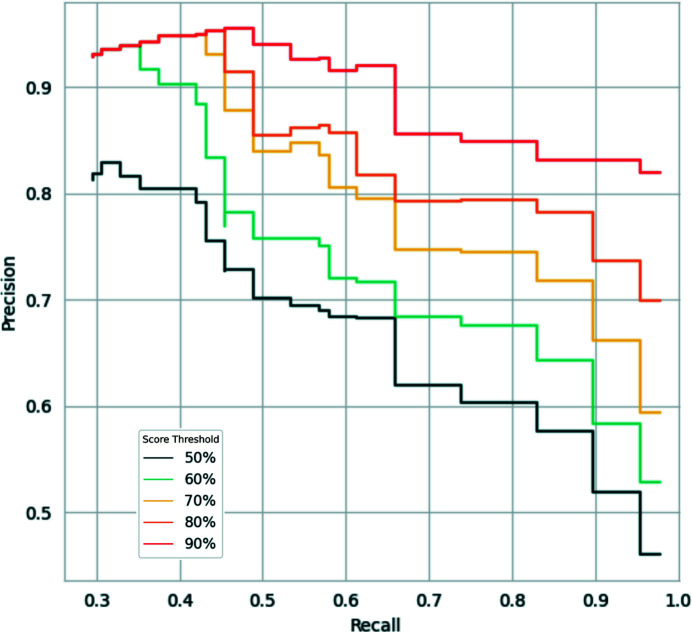
Precision–Recall curves obtained using different threshold values for the number of consecutive residues predicted as errors required to flag a possible register error in a model and the score required to classify a residue as an error. Curves coloured from dark blue to dark red correspond to predicted score threshold values of 50%, 60%, 70%, 80% and 90%, respectively. For each curve, Precision and Recall values were recorded after setting different thresholds on the number of consecutive errors required to flag an error: values start at 20 on the left and decrease down to a single residue on the right. Actual values are available in Supplementary Table S1.

The trained SVM classifier outputs a predicted likelihood that a given residue is within a model error (as defined in Section 2.1) on a residue-by-residue basis, meaning that it performs this prediction without any knowledge of the context of the residue of interest. In particular, it does not have any knowledge of the scores predicted for the neighbouring residues. Nevertheless, both sequence-register and other kinds of modelling errors are expected to span several consecutive residues within the model being validated. To exploit this expectation, values ranging from 1 to 20 were tested as threshold values for the number of consecutive residues predicted to be within an error. Different thresholds for the score required to predict a residue as an error were tested as well, with the following values being used: 50%, 60%, 70%, 80% and 90%. All combinations of these values were tested using the models in the EM modelling challenge data set, and the Precision and Recall values were recorded (Fig. 5 ▸). This revealed a negative correlation between the number of consecutive residues required to flag a possible model error and the Recall of these errors. Interestingly, higher Precision and Recall values were achieved as the score threshold increased, achieving a peak using a threshold of 90%. For instance, a threshold of three consecutive residues with a predicted score of at least 50% would achieve a Precision of 0.58 and a Recall of 0.9, while setting a threshold of 20 consecutive residues with a score of 50% or above would achieve Precision and Recall scores of 0.81 and 0.3, respectively. Depending on the use case, users may choose to maximize Precision over Recall or vice versa, which is why both thresholds can be tuned through the command-line option *conkit-validate*. Default values were set to a threshold of six consecutive residues with a score of 90% or higher to flag possible register errors, which were observed to achieve a Precision of 0.92 and a Recall of 0.66 on this test.

### 3.2. Contact-map alignment can be used for successful sequence reassignment of register errors

**Figure 6:**
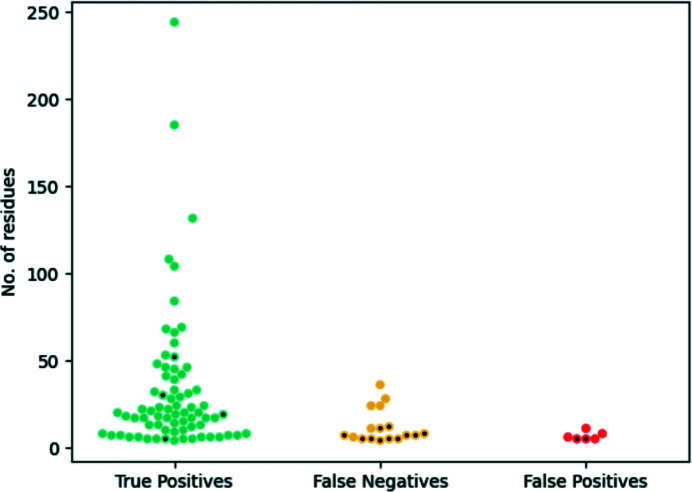
Performance of the sequence reassignment of register errors in the EM modelling challenges using contact-map alignment. The vertical axis shows the number of residues present in the register errors, which are represented with coloured points. Coloured points with a black dot represent register errors where residues had, on average, less than three predicted contacts. True positives (turquoise) are register errors where the correct sequence register was revealed after contact-map alignment (71 instances, of which four have less than three contacts per residue on average). False negatives (yellow) are register errors where no contact-map misalignment was detected at all (17 instances, of which 11 have less than three contacts per residue on average). False positives (red) are regions of the models where a contact-map misalignment was detected despite there being no register error (six instances, of which two have less than three contacts per residue on average). True negatives have been omitted for clarity; they consist of 6152 residues that are not involved in any kind of modelling error where no contact-map misalignment was detected.

In order to assess the performance of contact-map alignment as a method to reassign the correct sequence to register errors, all of the models submitted to the EM modelling challenges that form part of the training data set described in Section 2.1 were tested as follows. The contact maximum overlap (CMO) between the observed contacts in the submitted model and the predicted contact maps was calculated as described in Section 2.5. If the CMO for a given range of residues was achieved using a sequence register different to that observed in the model being validated (*i.e.* there is a misalignment between contact maps), then these residues were predicted to be part of a register error and the optimal sequence register was suggested as a fix. Encouragingly, of the 88 errors in this data set, 71 were detected using this contact-map alignment approach (Fig. 6 ▸). For all of these detected errors, the CMO between the predicted and the observed contacts was achieved using the correct sequence register, which was suggested as a fix, meaning that 87% of the errors analysed could have been fixed if this method had been available to the original authors of the models. Only 17 errors could not be detected using this method. Of these, only five errors were register errors, all of which had fewer than ten residues. The other 12 errors were non-sequence-register related modelling errors, for which this approach was unsurprisingly found to be unsuitable as an alternative sequence register cannot be found to achieve the CMO. Furthermore, only a small number of false positives were observed: instances where the contact-map alignment would suggest a different sequence register for the model despite there being no error. Six such cases were found, all of them consisting of misalignments that were less than ten residues in length. Regarding true negatives, a total of 6152 residues which were not involved in any kind of modelling error were also not involved in a contact-map misalignment. Additionally, the presence of sufficient contact information was revealed to be essential for the reliable detection of register errors using this approach: residues present in two thirds of the false negatives had on average fewer than three predicted contacts. Unsurprisingly, the absence of contact information hindered the detection of register errors in these instances. Similarly, two of the six false positives were observed to affect regions of the models where on average the residues had less than three predicted contacts. This revealed that a lack of sufficient contacts can also cause a contact-map misalignment to be inferred in cases where no actual register error is present, as it becomes difficult to find the optimal alignment between the maps. In contrast, only 5% of true positives were found to have less than three predicted contacts on average across the residues involved.

**Figure 7:**
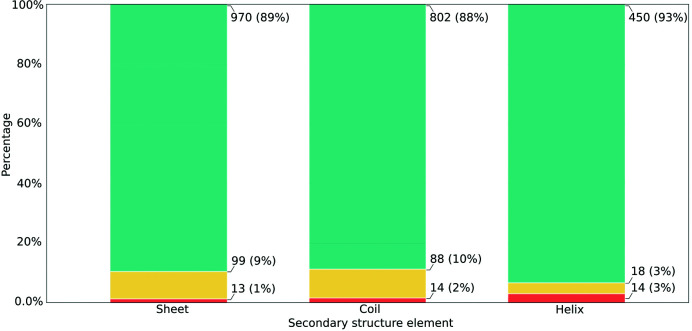
Secondary-structure context of residues found in register errors in models submitted to the EM modelling challenges. The vertical axis depicts the percentage of residues in each category. Residues are grouped along the horizontal axis depending on the secondary-structure element. Turquoise bars represent true positives (register errors where the correct sequence register was revealed after the contact-map alignment), yellow bars represent false negatives (register errors where no contact-map misalignment was detected at all) and red bars represent false positives (regions of the models in which a contact-map misalignment was detected despite there being no register error). Coloured bars are annotated with the total number of observations in each category and their corresponding percentage relative to the secondary-structure element. Secondary-structure assignment was performed using *DSSP*.

To further characterize those errors that could not be detected using this contact-map alignment approach, and those regions of the submitted models that were part of a contact-map mis­alignment despite there being no register error, an analysis of the distribution of residues across the different secondary-structure elements was carried out. Residues were assigned a secondary-structure element using *DSSP*, and the number of residues found in each category (true positives, false negatives and false positives) was recorded for each secondary-structure element (Fig. 7 ▸). Initial analysis of this distribution revealed that most register errors are located within β-sheets, followed by coils and lastly α-helices. Interestingly, there were no major differences in the distribution of residues between coils and sheets, with both having a high proportion of true positives and a significantly smaller proportion of false negatives and false positives. In the case of helices, the proportion of true positives was also high; however, the proportion of false positives was somewhat higher than in the other two cases.

### 3.3. Contact-map alignment and the SVM classifier are complementary methods for the detection of modelling errors

**Figure 8:**
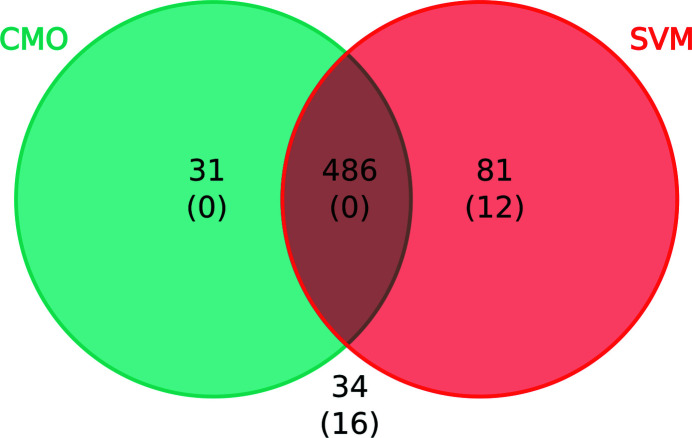
Venn diagram showing the number of residues found in modelling errors that could only be detected using the CMO approach (turquoise), the support-vector machines classifier (SVM, red), both methods (intersection) or neither of them (outside the circumferences). Residues found in the training set for the SVM classifier were discarded for this analysis. The numbers of residues involved in non-register-related modelling errors are shown in parentheses.

In order to assess whether the two proposed methods for model validation complement each other, an analysis of the residues found in register errors that could only be detected using one approach or the other was carried out. In order to do this, the 2428 residues found within register and other modelling errors in the EM modelling challenges were selected. Of these, 1943 residues had to be discarded as they were part of the data set used to train the classifier. This left 486 residues for which classification was attempted using the SVM classifier described in Section 3.1. The predicted class was then recorded for each residue, together with whether a different register was used to achieve the CMO. Encouragingly, most of these residues could be correctly identified as part of modelling errors using both approaches (Fig. 8 ▸) and only 34 residues were left undetected. Interestingly, while the calculation of the CMO proved to be an accurate approach for the identification of most residues found in register errors, there was a significant number of residues that could only be detected using the SVM classifier. Furthermore, only the SVM was able to identify any of the 28 residues originating from non-register-related errors found in this data set, detecting 12 of them. This suggests that while both approaches can successfully identify register errors, other kinds of modelling errors can only be found using the SVM, an indication that the methods complement each other.

### 3.4. Identification of register errors in cryo-EM structures deposited in the PDB

A new covariance-based model-validation pipeline was created based on combination of the trained SVM classifier and the contact-map alignment-based sequence-reassignment methods described in Sections 2.4 and 2.5. For each of the residues in the input model, the pipeline outputs the classifier’s predicted probability that the residue is part of a register error and whether a different sequence register is necessary to achieve the CMO in the contact-map alignment step. This pipeline was integrated into the Python package for the manipulation of covariance data, *ConKit*, in the form of a new command-line option *conkit-validate*. In order to assess the performance of the proposed pipeline, a large-scale analysis was carried out with the same data set as used to analyse the performance of the validation tool *checkMy­Sequence* (Chojnowski, 2022 ▸). This analysis localized possible register errors in 419 protein chains found across 246 structures deposited in the PDB with resolutions varying between 2.5 and 4.9 Å, all of which were determined using cryo-EM. Given computational restrictions, and the large size of some targets, it was possible to obtain *AlphaFold*2 predictions for 364 chains, representing 170 unique sequences, as described in Section 2.7. The amount of time required to produce these predictions varied between cases, depending mostly on the number of residues that were present in the sequence. Using the hardware described in Section 2.2, an average of 100 min was required to complete the *AlphaFold*2 predictions for a typical protein of 500 residues. Larger protein sequences required more computational time, requiring an average of 450 min for the prediction of sequences larger than 1000 residues. Faster predictions of similar quality may well be available in the future using the *MMseqs*2 API (Steinegger & Söding, 2017 ▸) as performed by *ColabFold* (Mirdita *et al.*, 2022 ▸).

**Figure 9:**
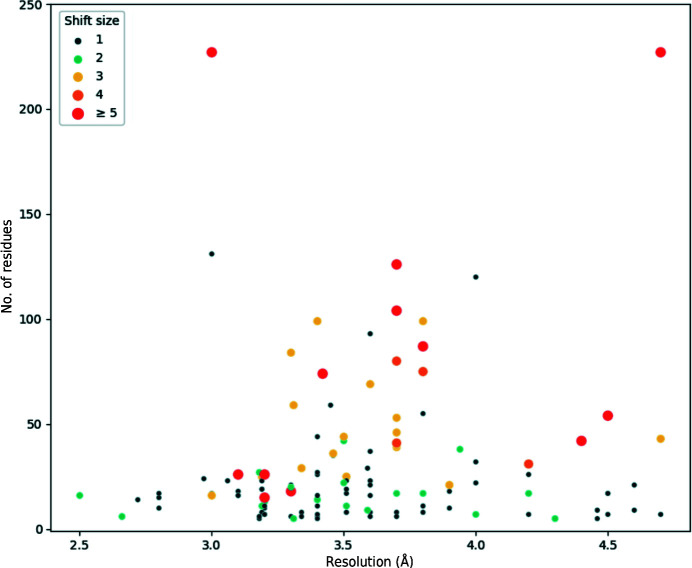
Characteristics of putative sequence-register errors found in the data set of cryo-EM structures deposited in the PDB. Each point represents a predicted register error found in a chain in the data set. Errors within five residues of each other were merged together into a single data point. For cases where errors are present in the same range of residues among several homomers, only one error is displayed. The vertical axis indicates the number of residues affected by the register error, which varies between five and 323 residues: one register error with more than 250 residues was found, but has been omitted for clarity. The horizontal axis shows the resolution, which varies between 2.5 and 4.9 Å. The colour and size of the point depict the average sequence shift observed in the error: one residue (dark blue), two residues (turquoise), three residues (yellow), four residues (light orange) and five residues or more (dark red).

The new *conkit-validate* pipeline was then used to analyse all of the chains in the data set, which revealed a total of 541 possible register errors for which the CMO was achieved using an alternative sequence register, of which 230 were found to be unique when taking into consideration the fact that some sequences are represented several times in the data set in homomeric structures. In order to discard instances where a contact-map misalignment was inferred despite there being no actual register error, the predicted errors were then filtered as described in Section 2.6. This decreased the number of predicted register errors from 230 to 130. Fig. 9 ▸ shows the characteristics of these 130 putative register errors.

While most register errors consisted of shifts of one or two residues affecting 50 or fewer residues, a total of 18 possible register errors consisting of 100 residues or more were found across 15 different structures (Table 2 ▸). Application of the criteria described in Section 2.6, which were designed to remove possible false positives, filtered out 11 of these large errors. Encouragingly, further inspection of these 11 errors revealed the presence of eight instances where the presence of a register error was unlikely and the contact-map misalignment could be explained due to a lack of sufficient contact information or high discrepancy between the deposited model and the predictions made by *AlphaFold*2. For two of the three register errors that did not meet the criteria but where no evidence of a false positive was found, an entry deposited at higher resolution in the PDB for the same protein and with the register that achieved the CMO was found. This is an indication that while the criteria proposed in Section 2.6 were effective in the removal of false positives, some true positives might also be removed in the process.

Further analysis of the seven large errors which met the criteria revealed for five of them the existence of at least one entry deposited in the PDB at higher resolution and with the register that achieved the CMO. Additional assessment of these models was carried out by calculating the Fourier shell correlation (FSC) between the affected range of residues and the density maps after 20 cycles of jelly-body refinement using *REFMAC*5 (Nicholls *et al.*, 2018 ▸). For each pair of models deposited for the same protein, this calculation was made using the same map deposited with the original structure where the possible register error was found. In the case of the deposited models with the alternative sequence register, the model was superimposed on the original structure using *GESAMT* with default parameters before refinement. Interestingly, in five out of seven cases the structure with the alternative sequence register achieving the CMO was also observed to achieve the highest FSC of the pair. While the FSC is a well established valuable metric for the agreement between the model and the map, it can sometimes be hard to interpret due to variations in local resolution or the effects of map sharpening. That this conventional model-to-map fit measurement does not support some of the models with the alternative register found to achieve the CMO may reflect these limitations.

Among the structures where an alternative deposition was found in the PDB with the proposed register, a 326-residue anti-CRISPR protein solved at 4.2 Å resolution (PDB entry 5xlp chain *D*; Peng *et al.*, 2017 ▸) was found to contain a possible register error corresponding to the entire structure having been shifted by ten residues towards the C-terminus. Interestingly, a structure of the same protein exhibiting the sequence register that achieved the CMO was deposited three years later at a resolution of 2.57 Å (PDB entry 6vqv chain *E*; Zhang *et al.*, 2020 ▸). Visual inspection of both models together with their respective EM maps revealed a clear improvement in the match between the model and the map in the later structure (Supplemetnary Fig. S1). The later, corrected structure was built *ab initio* and it seems that there was no mention of the incorrect register in the earlier structure.

### 3.5. *checkMySequence* and the proposed methods complement each other in the task of model validation

**Figure 10:**
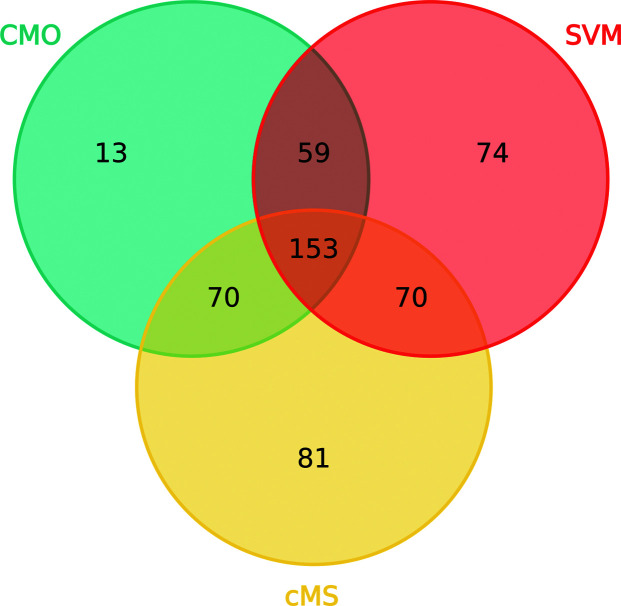
Venn diagram showing the number of predicted register errors found using *checkMySequence* (cMS; yellow), the contact-map alignment approach (CMO; turquoise) and the SVM classifier (SVM; red). Errors found using *checkMySequence* or contact-map alignment where the SVM predicted at least half of the residues to be part of modelling errors have been included in the intersection between the SVM and these methods. Any other stretch of at least six consecutive residues predicted by the SVM to be part of a modelling error with a probability of 90% was added as an error found by the SVM.

A comparison of the register errors found using the *checkMysequence* validation tool and the two proposed approaches was carried out in order to determine whether these methods complement each other. Predicted sequence-register errors found with each of these tools were recorded and a comparison was carried out (Fig. 10 ▸). Since the set of structures used to perform this analysis consisted of models in which *checkMySequence* found a possible register error during a previous study, a total of 374 predicted register errors were found across the 364 models in this data set, with every model having at least one error predicted by *checkMy­Sequence*. Using the combination of the contact-map alignment approach and the trained SVM, *conkit-validate* predicted the presence of 439 possible register errors for the same set of structures. Encouragingly, all three methods intersected in the prediction of 153 register errors, and 293 of the errors found with *checkMySequence* could also be found using either the contact-map alignment approach or the trained SVM. Additionally, the analysis revealed the presence of sequence-register errors that could only be predicted by either *checkMySequence* or by the combined use of the CMO and the SVM: 81 and 146 errors, respectively.

**Figure 11:**
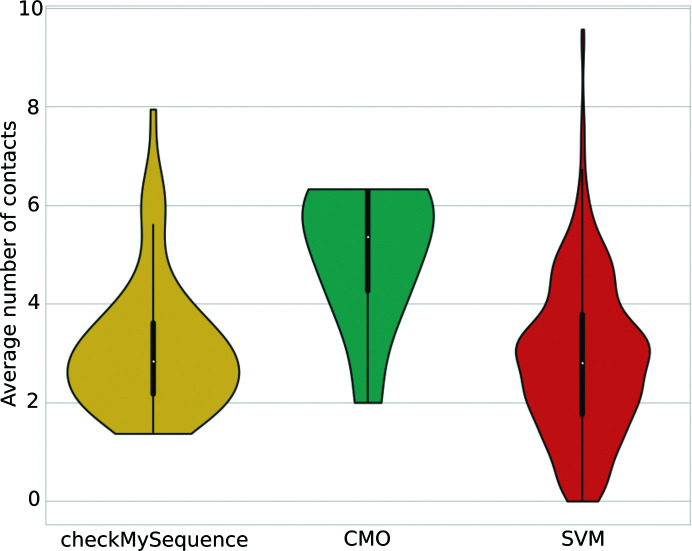
Distribution of the average number of contacts predicted for residues involved in sequence-register errors that could only be predicted by *checkMySequence* (yellow), the contact maximum overlap approach (turquoise) or the trained SVM (red). Black bars inside the violins depict the interquartile range, with a white dot showing the median and black whiskers showing the maximum and minimum quartiles.

Additional characterization of the errors that could only be found using one of the methods being compared was performed in the context of the available contact information (Fig. 11 ▸). This revealed that most of the register errors that could only be found using *checkMySequence* had a significantly lower number of contacts available than those that could only be found using contact-map alignment. This highlights the importance of sufficient available contact information for the reliable calculation of the CMO. In contrast, the trained SVM was able to detect register errors that contained fewer contacts than those detected with the CMO approach, a likely consequence of the fact that the most relevant feature, the wRMSD, was calculated using inter-residue distance predictions rather than contact predictions (Fig. 4 ▸). This helped *conkit-validate* close the gap with the number of errors detected by *checkMySequence* in cases where there is poor contact information: of the 151 errors that were detected by *checkMySequence* but not by the CMO approach, 50 were found to lack sufficient contact information to produce a reliable contact-map alignment, and of these 37 could be detected using the SVM.

These results highlight the existing synergy between these two approaches: while there might be errors that can only be detected with a map-based method such as *checkMySequence* or with a coordinate-based method such as *conkit-validate*, cases where these two independent approaches intersect can lead to the confident identification of sequence-register errors. While it is possible that errors with poor contact information will not be detected using *conkit-validate*, *checkMySequence* might still be able to detect them. Similarly, errors with poor map quality, poor local resolution or mistraced backbones proved to be harder to detect using *checkMySequence* in a previous study (Chojnowski, 2022 ▸), yet these factors should not diminish the performance of the CMO approach or the trained SVM.

### 3.6. Case study: register error found in a mycoplasma peptidase

Within the set of cryo-EM structures in which a possible register error was found using *conkit-validate*, a subselection of structures was made in order to assess whether it was possible to observe evidence of these errors when inspecting the model and the density map. In order to make this assessment as unambiguous as possible, structures solved at high resolution and that contain residues with aromatic side chains within the possible register error were selected. Among these structures, a mycoplasma peptidase deposited at 2.8 Å resolution was found to have a possible register error in one of its domains (PDB entry 7adk, chain *B*; Nottelet *et al.*, 2021 ▸). The function of this domain is not clear, although the original authors suggested that it is possibly a serine protease with a function related to the pathogenicity of the organism, specifically immune evasion.

**Figure 12:**
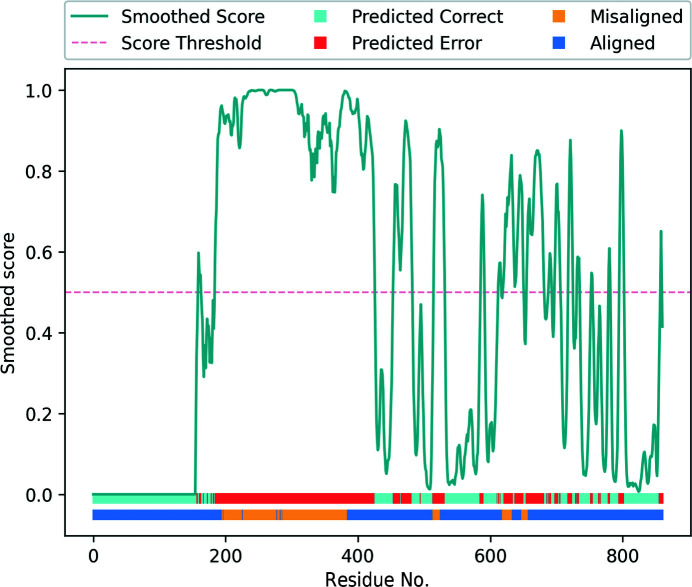
Validation report generated for PDB entry 7adk chain *B* using *conkit-validate*. Scores predicted with the SVM classifier are shown as a turquoise line and have been smoothed using a five-residue rolling average. The red dotted line shows the 0.5 score threshold. The top horizontal bar at the bottom of the figure shows for each residue position whether the predicted score was above (red) or below (cyan) 0.5. The lower horizontal bar at the bottom of the figure shows for each residue position whether the CMO was achieved using the sequence register observed in the model (dark blue) or an alternative register (yellow).

Analysis of the validation report produced by *conkit-validate* (Fig. 12 ▸) revealed four areas of the deposited model where the CMO was achieved using an alternative sequence register: residues 196–385, 515–524, 620–633 and 649–657. None of these errors could be verified by the existence of a different model deposited in the PDB with the register that achieved the CMO. The largest of these four errors corresponds to a 15-residue shift affecting 186 residues (residues 196–385). Interestingly, located among the residues at the end of this predicted register error, an unusual loop was found in the deposited model between residues 378 and 401. This loop was absent from the top-ranking model produced by *AlphaFold*2 (Supplementary Fig. S2), resulting in an otherwise structurally similar model with a different sequence register. Examination of the scores predicted by the SVM classifier for the other parts of the deposited model also revealed multiple stretches of residues predicted to be within modelling error. While most of these stretches coincided with the portions of the model where an alternative register was suggested using the CMO approach, high-scoring residues were also found in other areas of the model where the CMO did not indicate a potential register shift. Further inspection of the deposited model revealed that 25% of the residues predicted by the SVM to lie within modelling errors, despite there being no issues found using the CMO approach, were within 10 Å of at least five residues for which a different register was found using the CMO. This suggests that the predicted scores for these sets of residues could have been affected by neighbouring residues found within register errors. This can occur due to the nature of the metrics used as features for the SVM classifier: inter-residue contacts and distances of a correctly modelled set of residues can still be affected by incorrectly modelled neighbouring regions.

**Figure 13:**
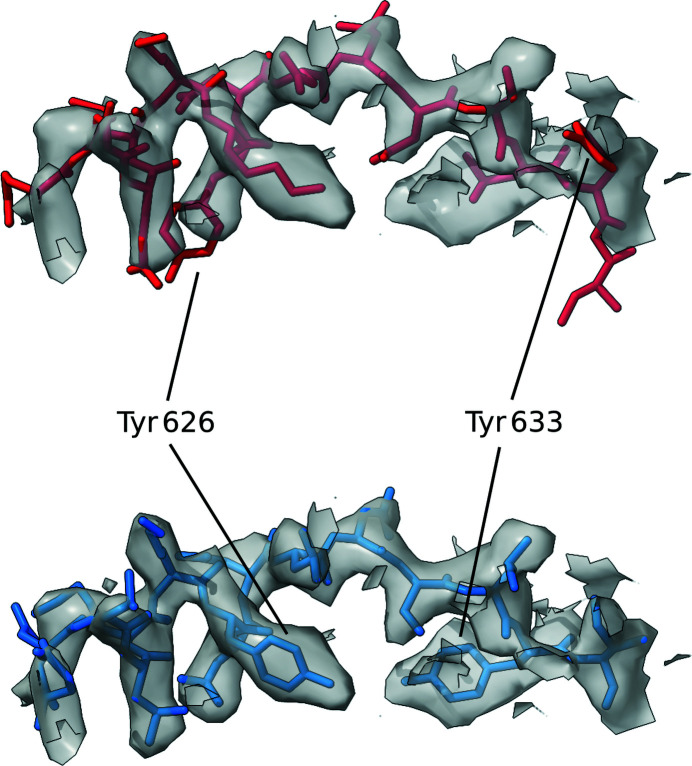
Detailed view of the section of the deposited model in which a possible sequence-register error was detected using *conkit-validate*. The density map is represented as a transparent grey surface and the level was set at 4.8σ. A mask of 3 Å around the model was applied. The original deposition is coloured red and the structure with the sequence register suggested by *conkit-validate* is in blue. Residues 626 and 633 have been highlighted for clarity. The error corresponds to PDB entry 7adk chain *B* residues 620–634.

Interestingly, analysis of the same structure using *checkMySequence* revealed a possible register error for residues in the range 613–637, which coincides with the set of residues in positions 620–633 where the CMO was achieved after shifting the sequence by one residue towards the C-terminus. Residues within this range were also assigned high predicted scores by the SVM classifier. Validation reports available for the PDB deposition showed that within this range of residues only Tyr633 was listed as a plane outlier and a rotamer outlier. Thus, the conventional validation metrics reported by the PDB did not flag any issue with this stretch. Visual inspection of the structure and the EM map was then carried out using *ChimeraX* (Goddard *et al.*, 2018 ▸), specifically for the range of residues in which both *checkMySequence* and *conkit-validate* predicted the presence of a register error. This region of interest was then reassigned to the new sequence register suggested by the CMO approach, using the sequence-shift tool available in *ISOLDE* (Croll, 2018 ▸). Visual inspection of these residues before and after applying this sequence shift revealed improvements in the model–map match, which is particularly evident when looking at residues with large side chains such as Tyr626 or Tyr633 (Fig. 13 ▸). Similarly, calculation of the FSC between the density map and this range of residues also revealed an improvement. After 20 cycles of jelly-body model refinement using *REFMAC*5 on both the original and the altered structures, FSC values of 0.65 and 0.73, respectively, were achieved, an indication that in this case the conventional model-to-map fit supports the alternative register.

### 3.7. Case study: register error found in an ion channel

Within the set of cryo-EM structures used to benchmark *conkit-validate*, the structure of a ligand-gated ion channel was found to have a possible register error in a set of residues located at the receptor ligand-binding domain. This structure was selected for further analysis in order to determine the presence of the detected register error as unambiguously as possible, due to the combination of high resolution (2.5 Å) and residues with aromatic side chains within the possible error (PDB entry 7l6q, chain *B*; Kumar *et al.*, 2021 ▸).

**Figure 14:**
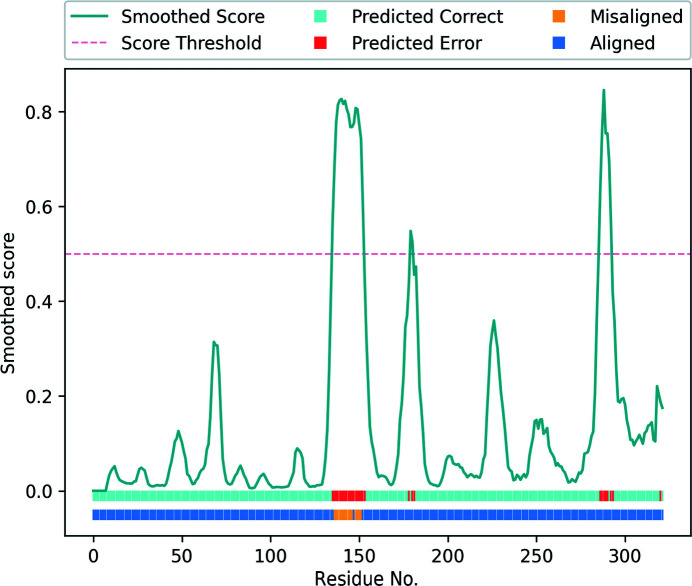
Validation report generated for PDB entry 7l6q chain *B* using *conkit-validate*. Scores predicted with the SVM classifier are shown as a turquoise line and have been smoothed using a five-residue rolling average. The red dotted line shows the 0.5 score threshold. The top horizontal bar at the bottom of the figure shows for each residue position whether the predicted score was above (red) or below (cyan) 0.5. The lower horizontal bar at the bottom of the figure shows for each residue position whether the CMO was achieved using the sequence register observed in the model (dark blue) or an alternative register (yellow).

Analysis of the validation report generated using *conkit-validate* for this structure (Fig. 14 ▸) revealed that the sequence register for residues in the range 137–152 had to be altered in order to achieve the CMO. Additionally, this same set of residues was predicted to be part of a modelling error by the SVM classifier, a further indication of a potential sequence-register error. While another 11 residues outside this range were also classified as part of modelling errors, none of these formed stretches of more than four consecutive residues, an indication that these are unlikely to be actual errors. Interestingly, analysis of the same structure using *checkMy­Sequence* revealed a possible register error for residues in the range 136–155, in agreement with the set of residues where a potential error was found using the other two methods. Examination of the validation report available for this PDB deposition revealed the presence of four rotamer outliers within this range of residues: Gln138, Gln139, Arg141 and Tyr148.

**Figure 15:**
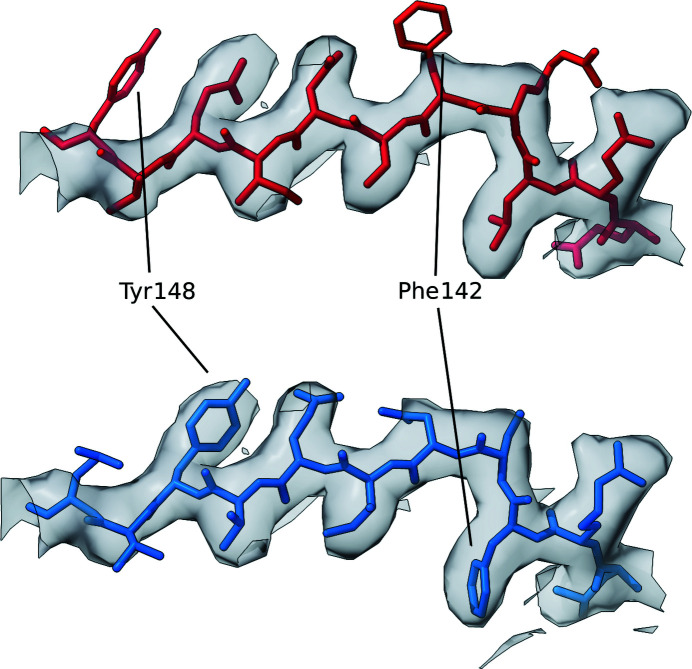
Detailed view of the section of the deposited model in which a possible sequence-register error was detected using *conkit-validate*. The density map is represented as a transparent grey surface and the level was set to 1.35σ. A mask of 3 Å around the model was applied. The original deposition is coloured red and the structure with the sequence register suggested by *conkit-validate* is in blue. Residues 148 and 142 have been highlighted for clarity. The error corresponds to PDB entry 7l6q chain *B* residues 138–148.

The sequence register for this range of residues was then shifted by two residues towards the C-terminus using *ISOLDE* in *ChimeraX* so that it would match the register that achieved the CMO between the predicted and the observed contact maps. Unexpectedly, after 20 cycles of jelly-body model refinement using *REFMAC*5 on both models, calculation of the FSC did not reveal an improvement in the model with the alternative register, with observed values of 0.84 and 0.83 for the deposited structure and the altered model, respectively. Despite this, there are several strong reasons to believe that the structure with the alternative register is more likely to be correct than the originally deposited structure. Firstly, visual inspection of the model before and after this modification revealed a clear improvement in the match between the side chains of these residues and the EM map (Fig. 15 ▸). Secondly, both the trained SVM and the CMO approaches predicted the presence of an error (Fig. 14 ▸). Thirdly, analysis of this PDB deposition, which consists of five identical chains, using *checkMySequence* suggested a possible sequence-register error for the same set of residues in three chains where an error was predicted using our methods. Since *checkMySequence* is a map-based validation tool, it is orthogonal to our methods. Finally, previous studies have observed that FSC can be inaccurate in the detection of issues affecting small regions of a model (Lawson *et al.*, 2021 ▸) such as this one. Taken together, this evidence suggests that the alternative register is indeed more likely to be correct.

Most of the structures in this benchmarking data set consist of structures solved using cryo-EM that were deposited at a time when no previous model of the same structure was available in the PDB. Interestingly, unlike most of the other structures in this benchmark set, this cryo-EM structure is of a ligand-gated ion channel that had already been solved by X-ray crystallography to a resolution of 3.3 Å in a previous study (PDB entry 2vl0, chain *B*; Hilf & Dutzler, 2008 ▸). Examination of this crystal structure revealed that it shares the same sequence register with the cryo-EM structure that was found to contain a potential register error. Visual inspection of this model together with the electron-density map also revealed similar features that could indicate a possible register error, particularly a poor model–map match for residues with large side chains. A better match was then achieved after modification of the deposited model using *ISOLDE* in *ChimeraX* to match the new sequence register proposed by *conkit-validate*, as revealed by further visual inspection (Supplementary Fig. S3). After 20 cycles of jelly-body model refinement using *REFMAC*5 on both the model with the alternative register and the original deposition, calculation of *R*_work_ and *R*_free_ also revealed an improvement, with observed values of 0.2172 and 0.2449, respectively, for the alternative structure, compared with scores of 0.2176 and 0.2485, respectively, for the deposited structure. Interestingly, an advanced search of the PDB to retrieve structures sharing at least 95% sequence identity with PDB entry 7l6q chain *B* returned hits for 33 chains, of which 16 shared the same (predicted erroneous) sequence register for this range of residues.

## 4. Conclusion

Here, we have presented new approaches for model validation based on the use of accurate inter-residue contact and distance predictions obtained using *AlphaFold*2. Firstly, a set of new metrics were fed into a support-vector machine classifier in order to train it to detect modelling errors based on the agreement or disagreement between the observed and predicted inter-residue distances. Trained using historical data from the EM modelling challenges, the classifier achieved an accuracy of 87% on the hold-out test set and proved to be capable of detecting modelling errors among structures deposited in the PDB. At this point it is worth noting that residues in the training and hold-out sets sometimes originated from the same register-shifted regions, which may have resulted in redundancy and overestimated accuracy. This could have been avoided by a more rigorous (and laborious) data stratification where each register-shift instance is represented in the training and test sets. We noted, however, that the number of support vectors defining the trained classifier is very low (757 of 3884 training observations), which is an indication of very good generalization properties (Cortes & Vapnik, 1995 ▸). If the low number of support vectors was the result of training-set redundancy and overfitting, the estimated test-set accuracy would be reduced compared with the training set. In our setup, however, we observed comparable accuracies in the training and test sets (87% and 86%, respectively). Contact-map alignment was then used to attempt sequence reassignment of possible register errors: parts of the model where the contact maximum overlap was achieved using a different register to that observed in the model were marked as possible sequence-register errors and the alternative register was proposed as a fix. Using this approach, it was possible to propose the correct sequence register for 87% of the register errors contained in a data set derived from the models submitted to the EM modelling challenges. We acknowledge that the performance may have been enhanced by the presence of some of the EM map modelling challenge structures and their homologues in the *AlphaFold*2 training set. Indeed, it has been observed that the *AlphaFold*2 confidence scores are higher for target sequences for which homologues are available in the PDB (Jones & Thornton, 2022 ▸). It is not clear, however, whether this bias comes from the direct use of templates, which can be accounted for relatively easily, or the availability of homologues in the training set. As a result, accounting for the *AlphaFold*2 training set bias would be very difficult: for example, the sequence-identity level threshold that would be set for the definition of an ‘unbiased’ test set is unclear. Contact-map alignment has previously been used in the field of *ab initio* modelling for the selection of templates among known protein structures (Ovchinnikov *et al.*, 2017 ▸), but to the best of our knowledge never in the context of correcting sequence-register errors. For the purpose of this study, the models used as the ground truth in these EM modelling challenges were considered to contain no errors. While it is possible that some errors could be present in these models, we believe this to be unlikely as only models with high-quality experimental data are used and exceptional care is taken when creating these models (Lawson *et al.*, 2021 ▸).

These two new approaches were combined together into a pipeline and integrated into *ConKit* as a new command-line option *conkit-validate*. Future work will incorporate the method into the *Iris* model-validation GUI that will soon be distributed with *CCP*4 (Winn *et al.*, 2011 ▸) and also *CCP-EM* (Burnley *et al.*, 2017 ▸) as part of new efforts to provide tools for map and model validation. In contrast to other approaches that compare the model coordinates with the density map derived from the experimental data (Pintilie *et al.*, 2020 ▸; Ramírez-Aportela *et al.*, 2021 ▸; Liebschner *et al.*, 2021 ▸; Joseph *et al.*, 2016 ▸), our approach relies solely on the use of deep learning-based inter-residue distance predictions, which are compared with the distances observed in the model of interest. Using this new pipeline, model validation was performed for a set of cryo-EM structures deposited in the PDB which were found to contain possible register errors using *checkMy­Sequence*. The results revealed that the use of the trained classifier in combination with contact-map alignment was successful in the detection and correction of 130 register errors which have inadvertently been deposited in the PDB. An inherent limitation of our proposed methods is the availability of high-quality inter-residue distances predicted by *AlphaFold*2. *AlphaFold*2 may, for example, struggle with cases in which only a shallow multiple sequence alignment is available, and special care should be taken in interpreting the results of our methods in these and any other cases where *AlphaFold*2 is not expected to perform well. While we do not have reasons to believe this was the case for the set of errors shown here, we acknowledge that in cases where *AlphaFold*2 predictions are of poor quality, such as in regions where the predicted model has a low pLDDT, these methods may falsely suggest potential register and modelling errors that do not really exist. In this and other respects, our method, being entirely coordinate-based, potentially has useful synergy with the map-based method of Chojnowski (2022 ▸): confident prediction of register errors can be performed in cases where these two independent methods intersect with each other. Together, they represent a new generation of software that can help to detect and correct the errors that even experienced structural biologists may inadvertently introduce when confronting the challenges of poorer resolution experimental data.

## Supplementary Material

Supplementary Figures S1-S3. DOI: 10.1107/S2059798322010415/qo5002sup1.pdf

Supplementary Table S1. DOI: 10.1107/S2059798322010415/qo5002sup2.xlsx

## Figures and Tables

**Table 1:** Accuracy, Area Under the ROC Curve (AUC), F1-Score, Precision and Recall achieved with the hold-out test set by support-vector machine (SVM), random forest (RF) and *k*-nearest neighbours (KNN) classifiers

Metric	SVM	RF	KNN
Accuracy	0.873	0.860	0.867
AUC	0.944	0.928	0.941
F1-Score	0.873	0.860	0.867
Precision	0.872	0.885	0.863
Recall	0.874	0.827	0.872

**Table 2:** List of register errors spanning 100 residues or more that were found using *conkit-validate* in the *checkMySequence* benchmark data set Structures listed under ‘Original Structure’ correspond to structures where an error was found using *conkit-validate* and those listed under ‘Alternative Structure’ correspond to a PDB deposition for the same protein where the register matches that found to achieve the CMO. The residue range and size of the register error might differ due to the presence of missing residues. FSC refers to the Fourier shell correlation between the specified range of residues and the density map. For each pair of structures, calculation of the FSC was performed using the map of the original deposited model where the error was predicted. In the case of the deposited models with the alternative sequence register, the model was superimposed on the original structure using *GESAMT* with default parameters before calculating the FSC. Sequence identity was calculated using all residues in both chains. The ‘Passes filtering’ column refers to whether the predicted sequence-register error meets the criteria described in Section 2.6 and provides a reason why these filters might not have been passed. Suspected false positives have been marked in the last column.

Original Structure									Alternative Structure						
PDB code, chain ID	Resolution (Å)	Year	Residues	Size	FSC	Citation	Passes filtering	Suspected false positive	PDB code	Resolution (Å)	Year	Sequence identity (%)	Residues	FSC	Citation
5nd8, *G*	3.7	2017	44–169	125	0.33	Khusainov *et al.* (2017 ▸)	Yes	No	6s0z, *F*	2.3	2019	100	44–169	0.45	Halfon *et al.* (2019 ▸)
5nd8, *O*	3.7	2017	27–130	103	0.54	Khusainov *et al.* (2017 ▸)	Yes	No	6s0z, *J*	2.3	2021	100	27–130	0.79	Halfon *et al.* (2019 ▸)
5xlp, *D*	4.2	2017	16–339	323	0.53	Peng *et al.* (2017 ▸)	Yes	No	6vqv, *E*	2.57	2020	100	32–357	0.74	Zhang *et al.* (2020 ▸)
5yz0, *A*	4.7	2017	2–547	534	0.49	Rao *et al.* (2018 ▸)	No: high Q-score	Yes	N/A	N/A	N/A	N/A	N/A	N/A	N/A
5yz0, *A*	4.7	2017	555–1520	720	0.53	Rao *et al.* (2018 ▸)	No: high Q-score	Yes	N/A	N/A	N/A	N/A	N/A	N/A	N/A
5yz0, *A*	4.7	2017	1884–2110	226	0.61	Rao *et al.* (2018 ▸)	Yes	No	N/A	N/A	N/A	N/A	N/A	N/A	N/A
6j5i, *b*	3.34	2019	3–208	205	0.34	Gu *et al.* (2019 ▸)	No: insufficient contact information	Yes	N/A	N/A	N/A	N/A	N/A	N/A	N/A
6klh, *A*	3.7	2020	1861–2007	107	0.20	Peng *et al.* (2020 ▸)	No: high Q-score	Yes	N/A	N/A	N/A	N/A	N/A	N/A	N/A
6rwa, *E*	4.0	2019	21–140	119	0.42	Leidreiter *et al.* (2019 ▸)	Yes	No	N/A	N/A	N/A	N/A	N/A	N/A	N/A
6uxv, *F*	4.7	2019	597–779	168	0.60	Han *et al.* (2020 ▸)	No: insufficient contact information	Yes	N/A	N/A	N/A	N/A	N/A	N/A	N/A
6vyh, *C*	3.0	2020	130–232	102	0.48	Pan *et al.* (2020 ▸)	No: high Q-score	No	5kvg, *L*	1.7	2016	96	118–211	0.64	Zhao *et al.* (2016 ▸)
6w2s, *2*	3.47	2020	644–870	226	0.43	Neupane *et al.* (2020 ▸)	Yes	No	6zp4, *C*	2.9	2020	98	644–870	0.56	Thoms *et al.* (2020 ▸)
6xe9, *A*	4.3	2020	852–1674	380	0.40	Yang *et al.* (2020 ▸)	No: insufficient contact information	Yes	N/A	N/A	N/A	N/A	N/A	N/A	N/A
6ybd, *v*	3.3	2020	33–154	121	0.33	Brito Querido *et al.* (2020 ▸)	No: insufficient contact information	No	6zp4, *E*	2.9	2020	98	33–154	0.09	Thoms *et al.* (2020 ▸)
6z6f, *D*	3.11	2021	117–235	118	0.46	Lee *et al.* (2021 ▸)	No: insufficient contact information	Yes	N/A	N/A	N/A	N/A	N/A	N/A	N/A
6zme, *Lt*	3.0	2020	7–138	115	0.15	Thoms *et al.* (2020 ▸)	Yes	No	7o7y, Bt	2.2	2021	100	9–138	0.005	Bhatt *et al.* (2021 ▸)
7adk, *B*	2.8	2020	196–385	184	0.36	Nottelet *et al.* (2021 ▸)	No: high Q-score	No	N/A	N/A	N/A	N/A	N/A	N/A	N/A
7kdp, *A*	3.6	2020	643–768	125	0.64	Kumar *et al.* (2021 ▸)	No: high Q-score	Yes	N/A	N/A	N/A	N/A	N/A	N/A	N/A
