# Supplementary material for: Using deep-learning predictions of inter-residue distances for model validation
Source: Acta Crystallogr D Struct Biol. 2022 Nov 25;78(Pt 12):1412–27. doi: 10.1107/S2059798322010415 (PMC9716559; doi:10.1107/S2059798322010415)
Supplement: Supplementary file 1 [file d-78-01412-sup1.pdf]

STRUCTURAL
BIOLOGY

Volume 78 (2022)

Supporting information for article:

Using deep-learning predictions of inter-residue distances for model validation

Filomeno Sánchez Rodríguez, Grzegorz Chojnowski, Ronan M. Keegan and Daniel J. Rigden

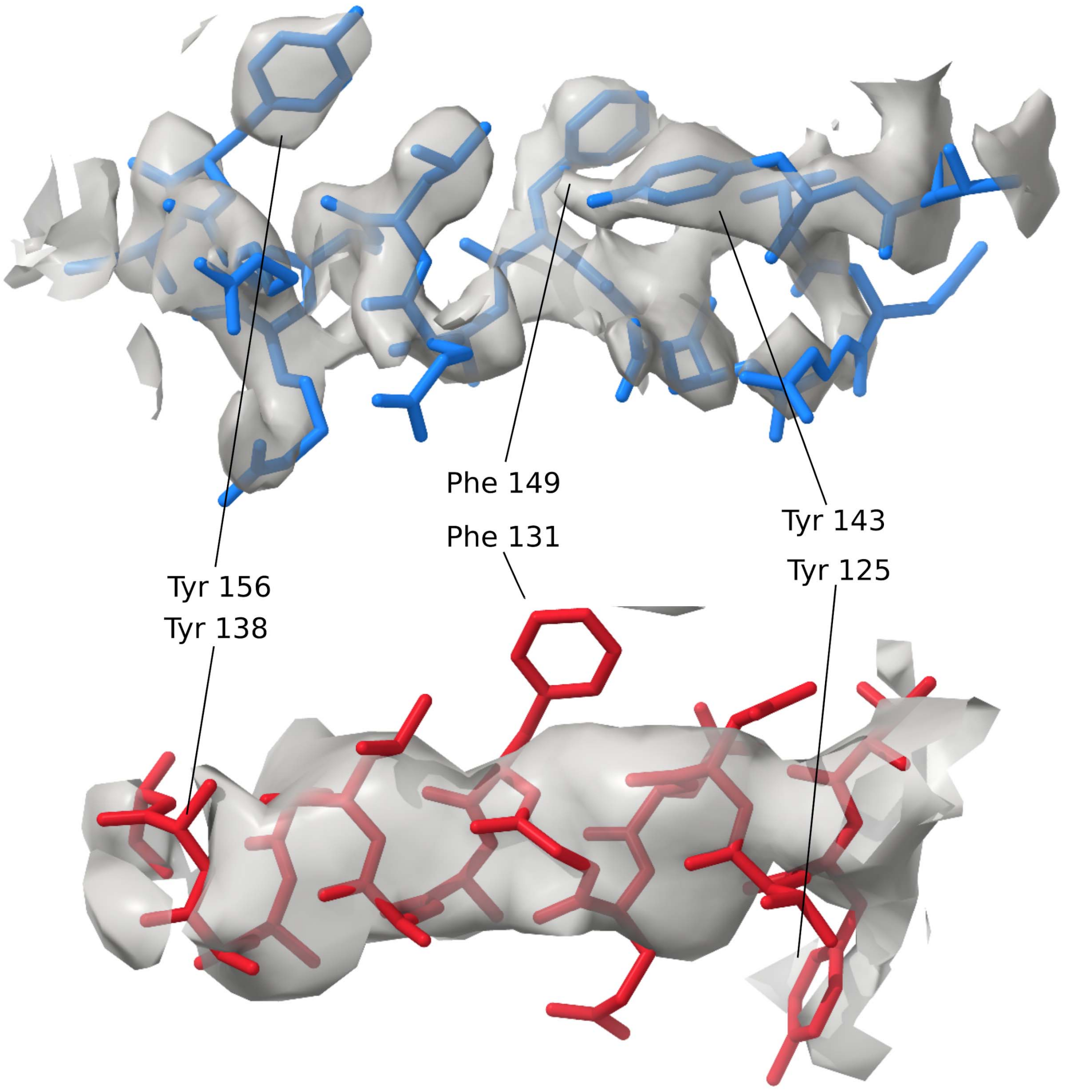

Figure S1 Comparison of the same section of 15 residues for a deposited structure (5XLP, chain D, resolution 4.7Å) where a possible 10-residue sequence shift was detected using conkit-validate (red model) and another deposited structure (6VQV, chain E, resolution 2.57Å) with the sequence register that achieved the CMO (blue model). The density map is represented as a transparent grey surface, and the level was set at 0.49σ for 6VQV and 0.113 for 5XLP. A mask of 3Å around both models was applied. Equivalent residues with large side chains have been highlighted for clarity (model residue numbering differs). No side chain was modelled in 5XLP for residue Tyr-138.

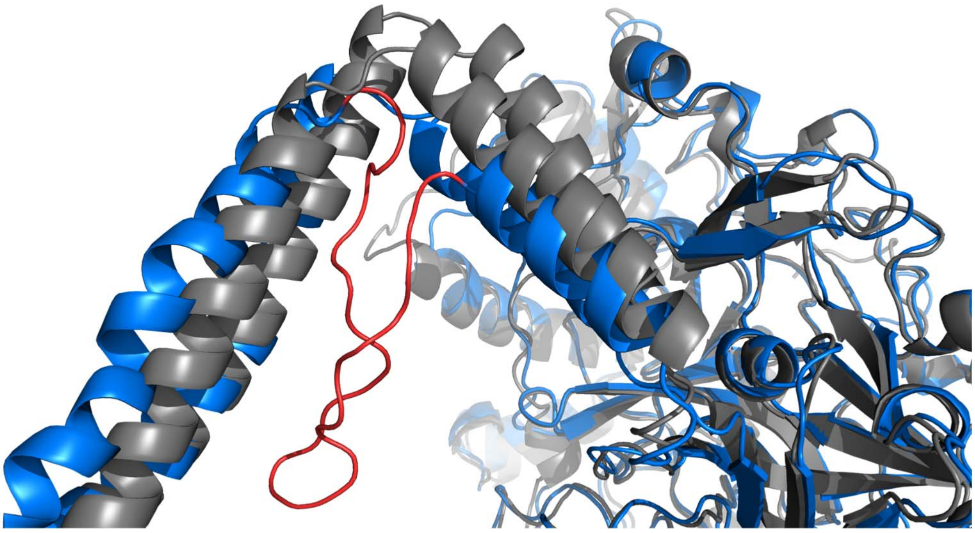

Figure S2 Comparison of deposited structure 7ADK:B (blue) with the top ranked model produced by AlphaFold 2 (grey). The image focuses at the loop found in the deposited structure for the range of residues between 378 and 401 which is coloured in red.

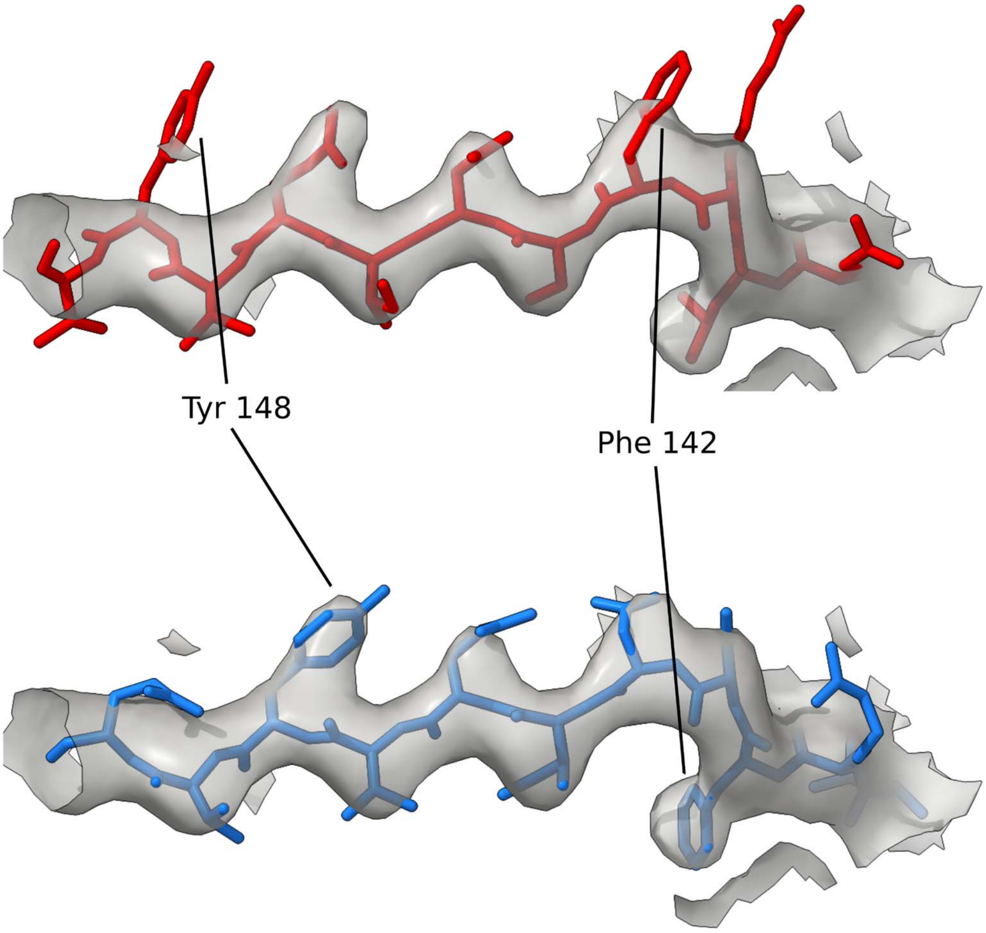

Figure S3 Detailed view of the section of the deposited model where a possible sequence register error was detected using conkit-validate. The density map is represented as a transparent grey surface, and the level was set to 1.06σ. A mask of 3Å around the model was applied. The original deposition has been coloured in red, and the structure with the sequence register suggested by conkit-validate in blue. Residues 148 and 142 have been highlighted for clarity. Error corresponds with PDB structure 2VL0, chain B, residues 139-149.
